# Can a two-hour lecture by a pharmacist improve the quality of prescriptions in a pediatric hospital? A retrospective cohort study

**Published:** 2017-12-15

**Authors:** Stephanie Vairy, Jennifer Corny, Olivier Jamoulle, Arielle Levy, Denis Lebel, Ana Carceller

**Affiliations:** 1 Department of Pediatrics, CHU Sainte-Justine, University of Montreal, Quebec, Canada; 2 Department of Pharmacy, CHU Sainte-Justine, University of Montreal, Quebec, Canada

## Abstract

**Background:**

A high rate of prescription errors exists in pediatric teaching hospitals, especially during initial training.

**Objectives:**

To determine the effectiveness of a two-hour lecture by a pharmacist on rates of prescription errors and quality of prescriptions.

**Methods:**

A two-hour lecture led by a pharmacist was provided to 11 junior pediatric residents (PGY-1) as part of a one-month immersion program. A control group included 15 residents without the intervention. We reviewed charts to analyze the first 50 prescriptions of each resident.

**Results:**

Data were collected from 1300 prescriptions involving 451 patients, 550 in the intervention group and 750 in the control group. The rate of prescription errors in the intervention group was 9.6% compared to 11.3% in the control group (p=0.32), affecting 106 patients. Statistically significant differences between both groups were prescriptions with unwritten doses (p=0.01) and errors involving overdosing (p=0.04). We identified many errors as well as issues surrounding quality of prescriptions.

**Conclusion:**

We found a 10.6% prescription error rate. This two-hour lecture seems insufficient to reduce prescription errors among junior pediatric residents. This study highlights the most frequent types of errors and prescription quality issues that should be targeted by future educational interventions.

## Introduction

A high medication error rate (5–24%) occurs in pediatric teaching hospitals, especially during initial training years of junior-residents.^1^–^3^ This has been recognized as an important cause of morbidity and mortality.^4^,^5^ Children are at higher risk of medication errors for many reasons including the need for weight-based dosing, medication dilutions, and environmental factors.^6^–^13^ Medication errors include prescription errors, transcription errors, errors in drug dispensing, medication administration errors, and errors attributable to patient compliance.^6^

Many interventions have been attempted to reduce medication errors:^14^ web-based education,^3^,^15^–^17^ preprinted order sheets,^18^–^21^ and computerized physician order entry, among others.^22^ Studies involving medical students have also demonstrated that various educational interventions can improve prescribing skills.^23^–^25^ However, few studies have focused primarily on the effectiveness of the course itself on prescription errors among pediatric trainees with encouraging data, but mitigated results.^3^,^16^,^26^,^27^ Literature is still too scarce to undertake a solid meta-analysis on the topic of targeting a high-quality educational intervention^26^,^28^–^30^ or even examining incidence of errors committed by junior doctors.^31^

In July 2013, we implemented an immersion program for residents beginning their pediatric residency training consisting of a one-month program with various pediatric clinics and wards, lectures, and department visits. This course included a lecture by a pediatric pharmacist on prescription error prevention. The aim of our study was to determine the effectiveness of this lecture integrated into the immersion program on the incidence of junior residents’ prescription errors and the quality of their orders.

## Methods

### Study design

This retrospective study was conducted at a large pediatric tertiary-care institution. Our Institutional Ethic Review Board approved the study. Participant informed consent was obtained prior to data collection.

#### Intervention group

In July 2013, post-graduate year (PGY) -1 residents beginning a pediatric residency-training program were exposed to the immersion rotation previously described. During this period, a pediatric pharmacist from our center provided a two-hour lecture explaining how to avoid medication errors. Teaching included a step-by-step approach to the medication circuit; pediatric drug safety, correct use of antibiotics in pediatrics and provision of resources were available for future reference. We collected prescriptions between the months of August and October 2013.

#### Control group

The control group included second year junior pediatric, first year pediatric neurology and genetics residents from the same center without the intervention. All prescriptions were reviewed during the first three months of their residency.

### Prescription collection

To minimize any confounding factors, we collected the first 50 prescriptions during the first three months of training for each resident in both intervention and control group.

To be included in the study, resident prescriptions were retrieved from the pharmacy information system (GesPharX8R) by using their license numbers. Patient files were accessed for each prescription. All prescriptions were handwritten. Two study investigators independently and retrospectively reviewed patients’ medical charts for data compilation. At our centre, once prescriptions are completed, pediatric pharmacists systematically review and validate all medication orders received at the pharmacy (average 550–750 prescriptions/day) to avoid medication errors.^15^,^32^

During the course of the study, participants were rotating on pediatric wards or specialties, in a level III neonatal intensive care unit (NICU) and in the emergency department (ED). All data regarding prescriptions were collected from patient charts, medication order sheets, and nursing notes. Demographic and clinical variables were recorded using a standard data collection sheet (Excel software 2011 for MAC version 14.4.5).

### Definitions

For this study, a prescription was defined as an inpatient written medication order. Verbal orders, such as those relevant to intravenous fluids, parenteral/enteral nutrition, and patient monitoring were excluded. Errors were considered in the absence of a documented dose, dose interval, specified route of administration, or a medication prescribed despite an identified allergy. In accordance with standard pediatric references, an incorrect dosage varying by 10% from the recommended dosing ranges was considered erroneous.^33^,^34^ Drug orders that did not require weight-based dosing were excluded from dosing error calculations.

The severity of prescription errors was classified according to the degree of clinical impact on patients, defined by the American Society of Hospital Pharmacist guidelines (ASHP),^6^ and previously described by Hartwig et al.^37^ For example, if no error occurred, it would classify as a level 0; if an error occurred that did not result in patient harm, it would classify as a level 1 (see Table 1). An intensive care status was given to patients who were transferred to the pediatric intensive care unit, or if the critical care team was consulted on the ward.

Quality errors had the potential to lead to a misinterpretation and eventually, to a medication error. Criteria were based on the ASHP^6^ and experts panel definitions.^35^,^36^ Absence of reported data such as allergy, weight, dose related to weight, date and hour of prescription, names of calculated drug when the drug formulas are composed, or the concentration in mg/ml, were classified as quality errors. Use of acronyms or trade names, presence of cross-out or ambiguity, use of a terminal zero, illegible handwriting or an unsigned order were also considered quality errors.

#### Statistical analysis

A two-proportion z-test was used to assess differences in prescription errors with a two-tailed test hypothesis. A Fisher test was used to assess differences in prescription quality errors between both groups. A p-value of ≤0.05 was considered significant. Statistical analyses were performed using GraphPad Prisme version 5.00 for Windows (San Diego, CA).

## Results

### Participants

All potential residents participating in the immersion program (11; intervention group) and all residents from the previous cohort (15; control group) were invited to participate in our study. All residents were recruited with a 100% participation rate. Demographic characteristics of both groups are represented in Table 2. All residents had previous pediatric exposure prior to the start of their residency (pediatric clerkship rotation). No differences were noted between groups.

### Prescriptions

A total of 1300 prescriptions involving 451 patients were reviewed - 550 from the intervention group and 750 from control group (Table 3). The majority of patients required a median of two orders per resident (range 1–15). Medications most frequently prescribed by both groups were antibiotics (34.0%), followed by acetaminophen/ibuprofen (16.8%). The complete list of medications is listed in Appendix A.

### Prescription errors

Prescription errors were found in 10.6% (n=138) of prescriptions, affecting 106 patients. A trend toward fewer errors was noted in the exposed-group (9.6%, 95% confidence interval [CI], 7.43 to 12.41) vs. control group (11.3%, 95% CI, 9.26 to 13.81), although this did not reach statistical significance (Table 3).

The most commonly encountered errors were dosing errors. In the intervention group, we found a lower rate of prescriptions with an overdose (p=0.04), but more orders with unspecified dosing (p=0.01). No statistically significant differences were noted for separate criteria such as dose intervals and routes of administration, or for 2 or 3 criteria combined. In our study, no medications were prescribed despite documented allergy. According to the ASHP classification,^6^ the severity of all reported errors reached level-1.

Description of prescription errors are shown in Table 4. The majority of errors occurred in patients younger than three months of age (6.1%, n=79). Although most prescriptions occurred on general pediatric teaching units, a larger proportion of errors occurred in the ED (25.0%, n=4/16), in the NICU (19.1%, n=41/215) and among critically ill patients (17.1%, n= 43/251). Among prescriptions prescribed during the holidays (n=13), there was an error rate of 23.1%. Prescription error orders most frequently involved antibiotic prescriptions (5.3% of all errors). Distribution of prescription errors was similar between both groups.

### Quality of prescriptions

We evaluated the quality of 1300 prescriptions (Table 5). We found no quality errors in 6.2% (95% CI, 4.46 to 8.51) of prescriptions in the intervention group vs. 4.4% (95% CI, 2.93 to 5.87) in the control group but the difference was not significant. The most frequent quality errors were unreported allergy status, unspecified dose/weight, use of acronym or trade names and unwritten weight on prescription sheets.

## Discussion

Overall, prescription errors occurred in 10.6% of prescriptions. It is difficult to compare error frequency from our study with those reported in other studies because the definition of prescription errors differs between studies, with frequencies varying between 0.005–24%.^7^,^21^,^38^,^39^ In the intervention group, we found fewer errors with lower rate of prescription above the recommended dose but more orders with unspecified unwritten dosing. This translates into 32 less errors in the exposed group during a three-month period. These results may reflect our population of prescribers and may not be generalizable to every hospital setting, in particular to hospitals with implemented electronic orders. However, this may be transferable to similar contexts.

Detecting significant differences in prescription error rates remains difficult given the small basal prescription error rate. A post-hoc analysis of the effect size calculated with the number of prescriptions within each groups shows that we were able to detect a 5% difference with a power of 90%. However, given that prescriptions were clustered (50 prescriptions/resident), this could have contributed to the lack of significant differences between groups for both prescription errors and quality of orders. To ensure enough power in future studies, more residents should be included considering a 5% difference as clinically significant.

The high frequency of prescription errors may be multifactorial. As previously reported in the literature,^38^,^40^–^42^ we found that dosing errors were the most frequent types of error and that patients younger than 3 months of age were also at greater risk of errors.^41^ Location in the ED and NICU was associated with the highest frequency of prescription errors, probably due to a high turnover of patients, critical situations and a stressful environment.^11^,^41^ We found an alarming frequency in prescription errors during holidays, but this represented a small number of the total number of errors. However, this phenomenon could be worth exploring in future studies. Antibiotics were the most prescribed drugs and also generated the highest rate of error.^9^,^39^,^43^

According to the ASHP classification,^6^ no errors resulted in patient harm. Indeed, they were all classified level-1 errors. Had we applied recently published NCCMERP taxonomy,^44^ our patients would have been classified as level B (error not causing patient harm) or C (some errors reached the patient). Given that 10.6% of prescriptions in our study contained errors affecting 106 patients, even if none resulted in patient harm, efforts should be made to reduce this rate even further. The short two-hour educational intervention included in a one-month immersion course, such as we used in our study, didn’t appear to be sufficient to reduce prescription errors among junior paediatric residents. However, we evaluated prescription errors for the three-month period following the intervention only; assessment of long-term skill retention should be considered in future research.

Pediatric prescription error is a major problem that warrants developing prevention strategies. However, this requires changes in hospital culture, collective motivation and inter-professional collaboration. Strategies should include developing various educational interventions and specific mandatory procedures when completing a prescription as detailed in Table 5 and by Ghaleb et al.^36^ Other examples include mobile applications or memory-cards, 21 additional training during medical clerkship,^23^ repetitive short tutorials to residents, pharmacy memos and individual prescriber feedback.^15^,^32^ In addition, we need to enable prescribers to adapt to their environment by overcoming constraint of time, overload, interruptions, and pressure from other staff.^6^,^7^ According to the current literature, complete prescription including reported allergy status, patient weight and time, contribute to decreasing medication errors. Moreover, an electronic order system could potentially decrease the high rate of quality errors by including helpful data and weight-based calculations.^22^,^45^

Study limitations include biases related to a retrospective chart reviews. In addition, our study may have been underpowered to detect significant results, given the small sample of residents. Strengths of our study included the 100% participation rate and the ability to record all identified errors with a high number of recorded items.

### Conclusion

Result from our study reveal that a short tutorial within an immersion program was insufficient to reduce prescription errors and improve the quality of orders among junior pediatric residents. This study also highlights the most frequent types of errors and prescription quality issues that should be targeted by future interventions and research.

## Figures and Tables

**Table 1:** Definitions of medication error severity levels^37^

Levels	Definitions
0	No medication error occurred
1	An error occurred but resulted in no harm to the patient
2	An error occurred that resulted in a need for increased monitoring of the patient
3	An error occurred that resulted in a need for increased monitoring and a change in vital signs but ultimately no harm to the patient
4	An error occurred that resulted in the treatment of an adverse event with another drug, increased the length of stay, or affected the patient’s participation in an investigational drug protocol
5	An error occurred that resulted in permanent harm to the patient
6	An error occurred that contributed to the death of the patient

**Table 2:** Participant demographics

		Intervention group 11 residents	Control group 15 residents
Institution where MD was obtained	McGill University	2	4
	University of Montreal	6	4
	Other Quebec universities	3	7

Gender	Female	10	9
	Male	1	6

Age range (years)	20–25	8	14
	26–30	3	1

Experience in general pediatrics (months)*	1	1	4
	2	9	7
	≥ 3	1	4

Experience in pediatric subspecialties (months)*	1	1	4
	2	7	9
	≥ 3	3	4

Degree obtained prior to MD	Undergraduate University College	2	0
		9	15

Residency program	Pediatrics	11	13
	Subspecialty**	0	2

* Before staring the residency

** Genetics or pediatric neurology

**Table 3:** Type of prescription errors and differences between each group

	Intervention group 550 prescriptions		Control group 750 prescriptions		p

	n	%	n	%	

**Errors**	53	9.6	85	11.3	0.32

Dose	23	4.2	30	4.0	0.44
Not-written	4	0.7	0	---	0.01
≤ 10 %	14	2.5	14	1.9	0.20
> 10 %	5	0.9	16	2.1	0.04
Dose Interval	17	2.4	34	5.0	0.09
Route of Administration	13	2.4	21	2.8	0.31
Medication prescribed despite chart-documented allergy	0	---	0	---	---
2 of the above criteria	2	0.4	7	0.9	0.11
3 of the above criteria	0	---	1	0.1	0.19

**Table 4:** Description of prescription errors between groups (n=138)

	Intervention Group 550 Prescriptions		Control Group 750 Prescriptions		Over 1300 Prescriptions	

	n	%	n	%	n	%

**Patient Age**						

≤ 3 months	33	6.0	46	6.1	79	6.1
> 3 months - ≤ 1 y.o.	3	0.5	4	0.5	7	0.5
> 1 y.o. – ≤ 5 y.o.	5	0.9	16	2.1	21	1.6
> 5 y.o. – ≤ 10 y.o.	4	0.7	4	0.5	8	0.6
> 10 y.o. – ≤ 18 y.o.	8	1.5	15	2.0	23	1.8

**Setting**						

General Pediatric						
Inpatients	23	4.2	43	5.7	66	5.1
Specialty Inpatients	11	2.0	16	2.1	27	2.1
Neonatal Intensive Care Unit	19	3.5	22	2.9	41	3.2
Emergency Department	0	---	4	0.5	4	0.3
*Intensive care status*	19	3.5	24	3.2	43	3.3

**Days of the Week**						

Monday–Friday	39	7.1	60	8.0	99	7.6
Saturday–Sunday	11	2.0	25	3.3	36	2.8
Holiday	3	0.5	0	---	3	0.2

**Class of Medication**						

Antibiotics	25	4.5	44	5.9	69	5.3
Acetaminophen/Ibuprofen	5	0.9	4	0.5	9	0.7
Pulmonary	1	0.2	8	1.1	9	0.7
Other Analgesics	4	0.7	2	0.3	6	0.5
Other treatments	18	3.3	27	3.6	45	3.5

y.o.: years old

**Table 5:** Description of quality errors between groups

	Intervention Group 550 Prescriptions		Control Group 750 Prescriptions		Over 1300 Prescriptions	

	n	%	n	%	n	%

Unreported Allergy	360	65.5	521	69.5	881	67.8
Dose related to weight not indicated	272	49.5	319	42.5	591	45.5
Use of acronyms or trade names for the drug	154	28.0	221	29.5	375	28.8
Weight not written	142	25.8	199	26.5	341	26.2
Cross-out or ambiguity	28	5.1	54	7.2	82	6.3
Undocumented date and time	11	2.0	36	4.8	47	3.6
Use of ≪ U ≫ for units	16	2.9	27	3.6	43	3.3
Inappropriate abbreviations	12	2.2	23	3.1	35	2.7
Route of administration not indicated	5	0.9	16	2.1	21	1.6
Name/dose of calculated drug not-written, when formula composed	12	2.2	6	0.8	18	1.4
mg/ml concentration not specified	6	1.1	3	0.4	9	0.7
Illegible handwriting	0	---	3	0.4	3	0.2
Unsigned order	0	---	2	0.3	2	0.2
Use of terminal zero	0	---	0	---	0	---

## Appendix A Drugs ordered by residents (intervention and control groups)

**Table:**

	Over 1300 prescriptions	%

Antibiotics	442	34.0
Acetaminophen/Ibuprofen	218	16.8
Vitamins, Supplements	89	6.8
Other analgesic or anti-inflammatory	88	6.7
Pulmonary	70	5.4
Corticosteroids	67	5.2
Hematologic treatments	58	4.5
Gastroenterological drugs	53	4.1
Neurologic treatments	51	3.9
Antiemetic, anti-histamines	32	2.5
Cardiologic drugs	30	2.3
Renal drugs	25	1.9
Benzodiazepine	24	1.8
Dermatologic treatments	18	1.4
Immunizations	15	1.2
Hormones, Contraception	13	1.0
Others	7	0.6
